# Challenges in the management of cardiovascular emergencies in Sub-Saharan Africa: a case report of acute heart failure complicating infective endocarditis in a semi-urban setting in Cameroon

**DOI:** 10.1186/s13104-018-3361-2

**Published:** 2018-04-25

**Authors:** Clovis Nkoke, Denis Teuwafeu, Cyrille Nkouonlack, Martin Abanda, Wilfried Kouam, Alice Mapina, Christelle Makoge, Ba Hamadou

**Affiliations:** 1 Buea Regional Hospital, Buea, Cameroon; 2 Clinical Research Education, Networking and Consultancy (CRENC), Douala, Cameroon; 3 Faculty of Medicine and Biomedical Sciences, University of Yaounde 1, Yaounde, Cameroon

**Keywords:** Infective endocarditis, Heart failure, Cardiac surgery, Case report, Sub-Saharan Africa

## Abstract

**Background:**

Infective endocarditis is a deadly disease if not promptly treated with antibiotics either in association with cardiac surgery or not. Cardiac complications are the most common complications seen in infective endocarditis. Heart failure remains the most common cause of mortality and the most common indication for cardiac surgery in patients with infective endocarditis which is increasingly available in resource limited settings.

**Case presentation:**

We report a case of native valve infective endocarditis of the aortic valve in a 27-year old female in a semi-urban setting in Cameroon complicated by severe aortic valve regurgitation and heart failure. She presented with a 2 month history of fever and a 2 weeks history of rapidly worsening shortness of breath. Emergency cardiac surgery was indicated which unfortunately could not be performed leading to the death of the patient.

**Conclusions:**

In spite of improvement in availability of diagnostic and therapeutic modalities for cardiovascular emergencies, affordability is still a challenge. Universal health coverage is advocated else the ravages of premature mortality from cardiovascular diseases may continue to remain unchecked in Sub-Saharan Africa.

## Background

Infective endocarditis (IE) is the most common and dangerous form of endovascular infection. It is a challenging diagnosis with a broad spectrum of presentations. Heart failure is the most important complication of IE which has the greatest impact on the outcome [1].

## Case presentation

**Fig. 1:**
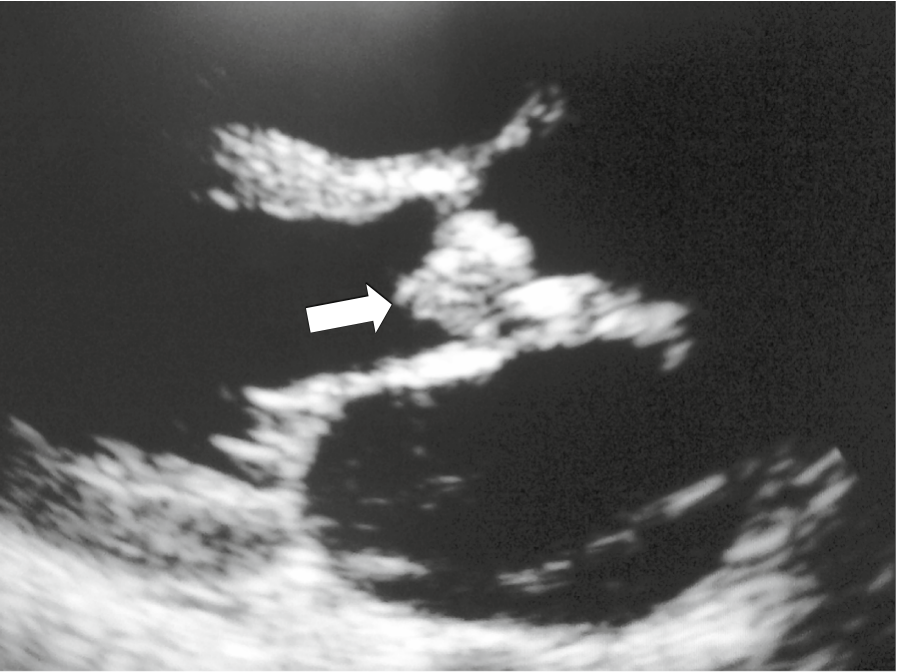
Transthoracic echocardiography showing vegetation (white arrow) on the aortic valve

A 27-year old female presented with a 2 month history of low grade fever, anorexia, fatigue and night sweats. Two weeks prior to admission, she developed rapidly worsening shortness of breath. There was no history of seizure or altered mental status. On examination, she was visibly short of breath (NYHA stage 4), lying at 45°. The respiratory rate was 36 cycles/min. The blood pressure was 130/40 mmHg in the right arm and 126/51 mmHg in the left arm. The heart rate was 110 beats per min. The temperature was 37.8 °C. The carotids were hyperdynamic, and there was a collapsing pulse. The heart sounds were regular, with a grade 3 early diastolic decrescendo murmur in the aortic area radiating toward the apex. There were bilateral basal lung crackles. There was conjunctival pallor. The neurologic examination was normal. Fundoscopy was not performed. A transthoracic echocardiogram revealed an oscillating mass on the aortic valve measuring 6 mm × 8 mm, compatible with a vegetation (Fig. 1). There was severe aortic regurgitation (PHT = 110 ms). The left ventricle and the left atrium were moderately dilated; the left ventricular ejection fraction was 71%. There was severe pulmonary hypertension (PASP = 61 mmHg). The diagnosis of infective endocarditis of the aortic valve complicated by severe aortic regurgitation was made. There was no evidence of a predisposing event. Blood cultures were not performed. The patient was started on intravenous diuretics, gentamicin and ceftriaxone and referred to the sole cardiac surgical center of the country for surgical treatment. However, financial limitations led to non intervention and the patient died 8 days following diagnosis.

## Discussions and conclusions

Over 80% of premature deaths due to cardiovascular disease occur in Sub-Saharan Africa. We have reported a case of infective endocarditis of the aortic valve in a young adult in Cameroon, complicated by severe aortic regurgitation and heart failure with an indication for cardiac surgery. The outcome was fatal.

Infective endocarditis is a deadly disease if not promptly diagnosed and adequately treated, either with antibiotics alone and/or in association with surgery [2]. The mortality rate approaches 30% at 1 year [3]. Early diagnosis of infective endocarditis requires a high index of suspicion with understanding of its risk factors, history and physical findings [2]. The clinical presentation of infective endocarditis can be acute or sub-acute. It is a disease not to be missed but the diagnosis can be challenging.

Complications are relatively common in infective endocarditis, which can be cardiac or extra cardiac. Heart failure is the most important complication of IE which has the greatest influence on the outcome. It was reported in 72% of patients with IE in a European series [1]. The occurrence of heart failure in IE is associated with high mortality [4, 5]. It represents the most common cause of death in native valve endocarditis and the most common indication of cardiac surgery for patients with infective endocarditis [1].

Early valve surgery in patients with heart failure is associated with a significant reduction in mortality compared with medical therapy alone [4]. The three main indications for early surgery in infective endocarditis are heart failure, uncontrolled infection and prevention of embolic events [6]. Cardiac surgery centers are scarce in resource constrained settings. Even when available, the high cost of cardiac surgery makes it unaffordable for many patients, especially in emergency situations.

Heart failure is usually the consequence of valvular regurgitation which may develop acutely as a result of perforation of a valve leaflet. Acute aortic regurgitation is poorly tolerated and usually rapidly progressive. Urgent surgery is indicated in this case regardless of the status of the infection. This suggests that our patient had a poor prognosis without surgery.

Our report highlights the challenges in the management of cardiovascular emergencies especially those requiring cardiac surgical modalities in resource limited settings. Our case had complications of infective endocarditis requiring cardiac surgery. However, financial constraints led to non intervention and subsequent death of the patient. Thus, advocacy for universal health coverage should be considered by stakeholders to curb premature mortality of cardiovascular diseases.

## Abbreviations

IE: infective endocarditis
PHT: pressure half time

## References

[1] Revilla A, López J, Vilacosta I, Villacorta E, Rollán MJ, Echevarría JR, Carrascal Y, Di Stefano S, Fulquet E, Rodríguez E, Fiz L, San Román JA (2007). Clinical and prognostic profile of patients with infective endocarditis who need urgent surgery. Eur Heart J.

[2] Paterick TE, Paterick TJ, Nishimura RA, Steckelberg JM (2007). Complexity and subtlety of infective endocarditis. Mayo Clin Proc.

[3] Cabell CH, Jollis JG, Peterson GE, Corey GR, Anderson DJ, Sexton DJ, Woods CW, Reller LB, Ryan T, Fowler VG (2002). Changing patient characteristics and the effect on mortality in endocarditis. Arch Intern Med.

[4] Nadji G, Rusinaru D, Rémadi JP, Jeu A, Sorel C, Tribouilloy C (2009). Heart failure in left-sided native valve infective endocarditis: characteristics, prognosis, and results of surgical treatment. Eur J Heart Fail.

[5] Vikram HR, Buenconsejo J, Hasbun R, Quagliarello VJ (2003). Impact of valve surgery on 6-month mortality in adults with complicated left sided native valve infective endocarditis: a propensity analysis. JAMA.

[6] Habib G, Lancellotti P, Antunes MJ, Bongiorni MG, Casalta JP, Del Zotti F, Dulgheru R, El Khoury G, Erba PA, Iung B, Miro JM (2015). ESC Guidelines for the management of infective endocarditis: the task force for the management of infective endocarditis of the european society of cardiology (ESC) endorsed by: European Association for Cardio-Thoracic Surgery (EACTS), the European Association of Nuclear Medicine (EANM). Eur Heart J..

